# R-based workflow to estimate chilling requirements in multiple fruit tree genotypes using Partial Least Squares regression: *Prunus armeniaca* L. case^☆^

**DOI:** 10.1016/j.mex.2025.103686

**Published:** 2025-10-26

**Authors:** Ana M. Muñoz-Morales, Germán Ortuño-Hernández, Juan Alfonso Salazar, Pedro Martínez-Gómez, Jose A. Egea, David Ruiz, Alvaro Delgado

**Affiliations:** Department of Plant Breeding, CEBAS-CSIC, P.O. Box 164, E-30100, Espinardo, Murcia, Spain

**Keywords:** Accelerated breeding, Chill models, Dormancy, Progeny evaluation, Pls, Temperate fruit trees

## Abstract

Accurate estimation of chilling requirements (CR) is essential for breeding and selecting temperate fruit trees adapted to specific agroclimatic conditions, particularly under global warming scenarios. Among the available methodologies to determine CR, the Partial Least Squares (PLS) regression procedure, based on long-term phenological and temperature records, offers a suitable approach to delineate the effective chill accumulation period. In this study, we present an R-based workflow developed using the agroclimatic functions integrated into the *chillR* package for R to determine the genotype-specific CR of 282 apricot (*Prunus armeniaca* L.) seedlings from two progenies grown in southwestern Spain. The pipeline generates standardized CR datasets suitable for downstream applications, including QTL mapping and the selection of promising genotypes for breeding purposes. This tool streamlines the estimation process, reducing the technical expertise and time required for CR estimation, thereby supporting efficient phenotypic selection and accelerating genetic research in temperate fruit trees. The complete code and associated datasets are freely available in a public repository (https://github.com/CEBASFruitBreed/R-workflow-ChillPLS), promoting the use across a range of temperate fruit species.

• Uses long-term flowering observations and temperature records to determine genotype-specific chilling requirements.
• Integrates PLS regression procedure within an R-based workflow to estimate chilling requirements from datasets comprising multiple genotypes.
• Generates standardized outputs suitable to support genetic analysis and informed breeding decisions.

## Graphical abstract

**Figure:**
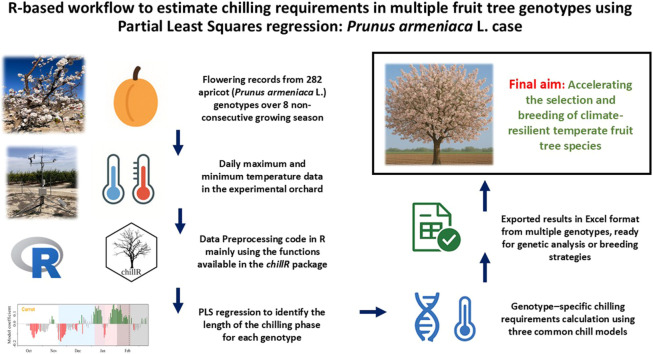

Specifications table

**Table:** 

**Subject area**	
**More specific subject area**	Chilling requirement estimation in temperate fruit trees for large datasets
**Name of your method**	ChillPro: Evaluation of chilling requirements in large fruit trees progenies
**Name and reference of original method**	N/A
**Resource availability**	Related R code is available in: (https://github.com/CEBASFruitBreed/R-workflow-ChillPLS)

## Background

Fruit breeders routinely generate large segregating progenies each year through controlled cross-pollinations. These progenies are monitored over multiple seasons for key phenotypic traits such as flowering time, to identify promising individuals for commercial release as new cultivars or use as potential parents in further breeding cycles. Additionally, biparental populations are evaluated to construct genetic maps and identify loci associated with important agronomic traits [1]. For temperate fruit trees, one of these traits is chilling requirements, especially important for the selection of new varieties in a context of climate change. The trees spend the winter months in a dormancy phase that allows them to survive unfavorable climatic conditions [2,3]. To break dormancy and ensure a good and uniform flowering in spring, each cultivar must meet its genotype-specific chilling requirement (CR). Consequently, the careful selection of cultivars well adapted to specific growing regions where it can fulfill its CR every year has become increasingly important for sustaining the profitability of fruit production [4,5]. Traditionally, CR in *Rosaceae* species such as apple, pear, almond, peach, sweet cherry, and apricot has been estimated using a labor-intensive and time-consuming forcing method [6,7]. This approach involves collecting shoots weekly and exposing them to controlled environmental conditions for a fixed period of time, limiting its use to a relatively small number of genotypes each year. As an alternative, statistical methods based on using long-term field observations of flowering dates and temperature records offer promising outcomes. Among them, Partial Least Squares (PLS) regression has been widely used to define the chilling period and estimate genotype-specific chilling requirements [[8], [9], [10]]. Although PLS regression can produce robust results depending on the species, location, and availability of phenological data [6], applying PLS regression to large datasets remains time-consuming since two separate steps are needed. First, determining genotype-specific chilling periods and second calculating CR values within those periods for numerous individuals. To address this challenge, an R script, using functions from the *chillR* package [11], and its corresponding workflow have been developed to automate this process. This code enables an efficient estimation of CR across large datasets of flowering records collected by breeders or researchers over recent decades. The final output is produced in a format ready for downstream molecular analyses, such as QTL mapping. This automated pipeline significantly accelerates the generation of CR data in breeding programs, supporting both phenotypic selection and genetic research. In this paper, we use as a case study the flowering records collected by the CEBAS-CSIC Fruit Breeding Team in Cieza (Murcia, southwestern Spain; 38° 16′ N, 1° 16′ W, 241 m a.s.l.) over eight non-consecutive growing seasons for two F1 apricot (*Prunus armeniaca* L.) progenies. These progenies resulted from the crosses ‘Bergeron’ × ‘Currot’ (‘*B* × C’) and ‘Goldrich’ × ‘Currot’ (‘*G* × *C*’), comprising 126 and 156 seedlings, respectively.

## Method details

Following this introduction, we present the code and workflow used in our case study. The code, written in the R programming language, outlines the essential steps required to generate the outputs and is accompanied by a brief explanation of the results and their implications. All dormancy-related metrics, including the PLS regression analysis and the calculation of CR using the three most common chill models, are implemented in the *chillR* package (version 0.76) [11]. The complete code and dataset used in the analyses described below are freely available in the following GitHub repository (https://github.com/CEBASFruitBreed/R-workflow-ChillPLS).

### R libraries

To run the functions used in this workflow, the following R libraries must be loaded prior to execute the script: '*tidyr*', '*openxlsx*', '*readxl*', '*dplyr*', '*chillR*', '*stringr*', and '*tools*'.

### Data preprocessing workflow

To conduct the analysis, flowering data, defined as full bloom (∼50 % of flowers open) [12], must be expressed in Julian dates (day of the year; DOY) and organized in an Excel file. Each genotype should have a single DOY value per growing season (see Table 1). The dataset must be structured with columns arranged from left to right, starting with “Genotype,” followed by columns labeled ‘BD12’ to ‘BD25,’ each representing a specific year in the 2000s for which flowering data were available in this case study. Each row corresponds to a unique genotype, identified using coded names.

**Table 1:** Example of the Excel file structure used for flowering date input in the ‘*G* × C’ population.

Genotype	BD12	BD13	BD14	BD21	BD22	BD23	BD24	BD25
1_1	*	75	69	71	81	83	80	85
1_2	70	53	53	60	63	72	59	69
1_3	72	63	62	64	72	80	75	76
1_4	72	63	63	64	67	72	68	75

The code below illustrates how to import and preprocess two datasets containing blooming dates, each corresponding to controlled hybridization crosses of apricot cultivars (‘BxC’ and ‘GxC’). The data are read from an Excel file using the *read_Excel* function, with each bi-parental population stored in a separate worksheet. To ensure data quality, any records marked with an asterisk (*), which indicates very poor flowering and the inability to assign a reliable date under field conditions are treated as missing values and removed using the *na.omit* function, which eliminates rows with incomplete or invalid entries. While missing data can be handled according to user preference and potentially removed before starting the analysis, in this code the symbol “*” is specifically used to exclude such observations. The same procedure would apply analogously for the GxC population.



The next step consists of reformatting the previously loaded dataset to match the structure used in the CEBAS-CSIC flowering records. This step can be skipped if the phenology dataset has already been prepared following the format shown in Table 2. In our example, we reformatted the dataset using the *process_data_format* function, specifically designed to prepare and filter the data for downstream analyses. This function uses the *pivot_longer* function from the *tidyverse* package to convert the dataset from wide to long format, collapsing all columns starting with "BD" (bloom dates) into two new columns: “Year”, which transforms the original column names by replacing the “BD” prefix with “20″ to generate a four-digit year (e.g., “BD12” becomes “2012″), and “pheno”, which contains the flowering date values for each year. Finally, the reformatted dataset is saved as an Excel file (Table 2) for subsequent analyses.

**Table 2:** Flowering records of the ‘BxC’ population formatted in Excel for analysis using the *chillR* package.

Genotype	Year	pheno
1_1	2012	74
1_1	2013	69
1_1	2014	62
1_1	2021	60
1_1	2022	67
1_1	2023	79
1_1	2024	75
1_1	2025	83
1_4	2012	78
1_4	2013	65
1_4	2014	63
1_4	2021	64
1_4	2022	65
1_4	2023	83
1_4	2024	71
1_4	2025	74



The *load_climatic_file* function is designed to process temperature data for estimating cultivar-specific chilling requirements. The workflow begins by evaluating data completeness using the *check_temperature_record* function, which identifies any missing values (NAs). Temperature records are then filtered starting from one year prior to the earliest available phenological observation. For this period, temperature values are corrected using the *fix_weather* function. The dataset is subsequently transformed into hourly temperatures using *stack_hourly_temps*. Based on the latitude of the experimental orchard, hourly values are reconstructed from daily minimum and maximum temperatures following the methods of [13] and [14]. Daily chill accumulation is then calculated using the *daily_chill* function, which returns a list containing validation results, the number of missing values, the corrected temperature dataset, and the computed daily chill. This workflow is demonstrated using the temperature dataset “Cieza11–25.xlsx” (Table 3) which includes daily maximum and minimum temperature at latitude of 38.11°.

**Table 3:** Structure of the Excel file used for daily temperature data with columns representing the main variables used in the analysis: Day (day of the month), JDay (Julian day, indicating the day of the year), Month (calendar month), Year (calendar year), Tmax (daily maximum temperature in °C), and Tmin (daily minimum temperature in °C).

Day	JDay	Month	Year	Tmax	Tmin
1	1	1	2011	14.46	6.62
2	2	1	2011	15.72	6.76
3	3	1	2011	15.32	3.36
4	4	1	2011	15.17	0.42
5	5	1	2011	14.01	2.80
6	6	1	2011	20.34	3.85



The *process_genotypes* function is designed to organize genotypic data into subsets and export each as a separate Excel file. From the Genotype column, two numerical groups are generated: group1, which corresponds to the number before the underscore symbol (_), and group2, which identifies the number after the underscore. These identifiers are used to group the data with *group_by*, and the records are then split into distinct subsets using *group_split*. Each subset is saved as an individual Excel file, with filenames that include the provided identifier (id) along with the corresponding group1 and group2 values. This function simplifies data organization, facilitating downstream management and analysis. In this example, it was applied to the files BxC_edit_filt.xlsx and GxC_edit_filt.xlsx, resulting in multiple output files grouped by genotype combinations.



### PLS-based workflow for chilling requirement analysis

**Fig. 1:**
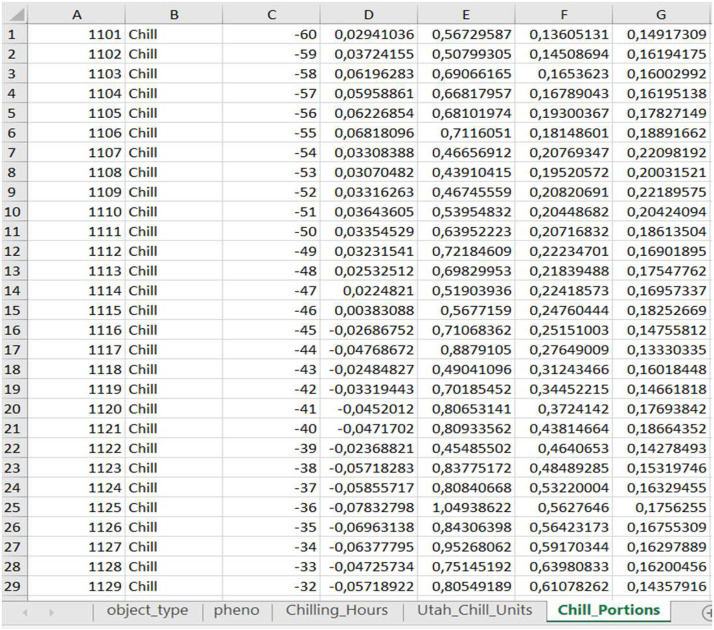
Results of the chilling phase obtained by applying PLS regression. The third column indicates the day of the year (DOY) within the growing season, with negative values representing the number of days remaining until the end of the year. Columns 4 and 5 show the Variable Importance in the Projection (VIP) scores and the corresponding model coefficients, respectively. Columns 6 and 7 represent the daily mean and standard deviation of the selected chilling metric (Chill Portions in our study case).



This section describes the automated processing of multiple Excel files containing flowering data to perform a PLS regression analysis. It begins by generating a list of files located in the “Genotypes/” directory that match a specific naming pattern. Each file is read iteratively, extracting its content and base name while removing unnecessary columns (group1 and group2) and adjusting key data types (e.g., converting Year to integer and pheno to factor). The *PLS_chill_force* function is then applied to each dataset, using preprocessed climate data and setting the start of the dormant season to November (split_month = 11). This starting month was selected based on previous regional studies showing that chill accumulation typically begins in November [15,16]. Across three consecutive seasons in these studies, the first chill portion was observed between November 4th and November 8th. The output of each analysis is saved as a new Excel file (see Fig. 1) in the “Genotypes/Results_PLS_Genotypes/” directory, with filenames corresponding to their source files to ensure organized tracking and easy retrieval. For each genotype, the function extracts group identifiers from the Cultivar column, splits the dataset into subsets based on unique group combinations, and exports each subset as a separate Excel file. This approach ensures that subsets are well-organized and readily accessible for further analysis. Chilling accumulation is calculated using three widely applied models in horticultural research: the Chilling Hours Model, the Utah Model, and the Dynamic Model. We strongly recommend using the Dynamic Model [17,18], as it has been shown to produce more reliable results in regions characterized by mild winter conditions [19,20].

The *process_PLS_file* function is designed to handle multiple datasets containing daily chill values (e.g., Chill Portions) and extract key information on the chilling period associated with each genotype’s entrance and release from endodormancy. This function applies to the PLS regression approach, which determines the chill accumulation window based on two primary outputs: the Variable Importance in Projection (VIP) scores and the model coefficients.

Following the criteria established by [21], the endodormancy phase is identified by a series of consecutive days with negative model coefficients and VIP scores equal to or greater than 0.8. For each dataset, the genotype identifier is retrieved, and rows are filtered to retain only those that meet both conditions. The function then defines the chilling period for each genotype by identifying the first and last day within this filtered range. Because the PLS approach accounts for the overlap between chilling and heat accumulation phases, and the current consensus among dormancy-related researchers [22], the code restricts chill accumulation to end maximum one day before the genotype’s median bloom date. The resulting data is then compiled into a single data frame and exported as an Excel file, facilitating the integration of climatic and phenological data for subsequent analyses.



The code below integrates phenological and climatic data for two apricot progenies, ‘BxC’ and ‘GxC’, using the *process_PLS_file* function. It begins by retrieving lists of Excel files containing Chill Portions data for each genotype from their respective directories. The function is then applied to both datasets, extracting the chilling period information for each genotype and saving the results into separate Excel files for streamlined analyses.



The *process_Jday* function calculates temperature-based metrics for each genotype in a dataset, with a primary focus on chill accumulation. For every genotype, the function determines the starting Julian day (Start_JDay) based on the provided daily values and processes the corresponding hourly temperature data using the *stack_hourly_temps* function. It then applies the *tempResponse* function to estimate chilling responses according to several chill models. The results for each genotype are stored in a structured list and exported as individual Excel files, named using the corresponding genotype and group ID. This approach enables a detailed, genotype-specific assessment of chilling requirements across multiple genotypes.



The *process_final_file* function processes multiple Excel files containing phenological data and extracts chilling metrics for selected seasons. It begins by filtering the data to retain only the predefined seasons of interest. Next, a year-based suffix is then extracted to identify each season, and the corresponding genotype is assigned based on the file name. The function dynamically reshapes the data using pivot tables, generating separate columns for each season, each containing the three chill metrics used in the analysis: Chill Hours, Chill Units and Chill Portions. Finally, the results from all files are compiled and saved into a single Excel file, which serves as the final output of the workflow.



**Fig. 2:**
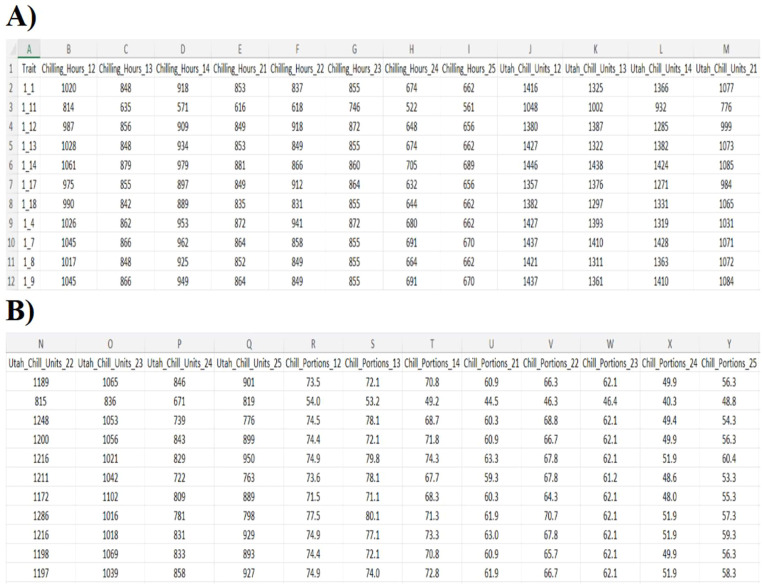
Final output summarizing chilling accumulation for each apricot genotype in the BxC population across multiple seasons. The table presents values from three chilling models—Chill Hours, Chill Units, and Chill Portions—for each genotype and growing season. This standardized output facilitates the comparison of chilling requirements across years and genotypes, supporting downstream genetic and phenological analyses.



In the final step of the workflow, all Excel files from the specified directories are read, relevant data are filtered, and seasonal chilling metrics are extracted. The processed results are then compiled to generate a final output file for each genotype. This step ensures a standardized, automated, and reproducible process for summarizing seasonal chill metrics across all genotypes (Fig. 2).

### Method validation

**Fig. 3:**
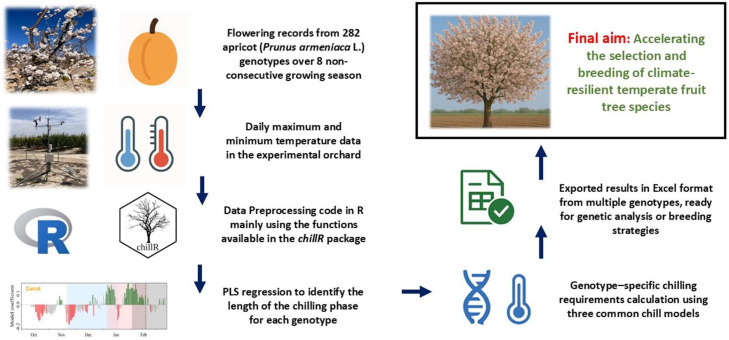
Graphical summary of the workflow, showing input files (flowering and climate data in Excel files), intermediate steps (preprocessing and PLS regression analysis), and the final outputs (genotype-specific chilling requirement estimates).

In fruit tree breeding, phenotyping is often a major bottleneck, especially for traits linked to dormancy release and flowering. Determining cultivar-specific CR is particularly important, since this trait defines cultivar adaptability to specific climates under current and future conditions. Traditionally, CRs are estimated using the forcing shoots method, which involves collecting dormant shoots during winter and placing them under controlled conditions (20–25 °C) to induce bud break. Although widely used, this approach is labor-intensive, time-consuming, and unsuitable for large breeding populations. The methodology presented here provides an efficient alternative, allowing breeders to rapidly process and analyze extensive phenological datasets and generate standardized CR values for downstream applications (Fig. 3).

**Fig. 4:**
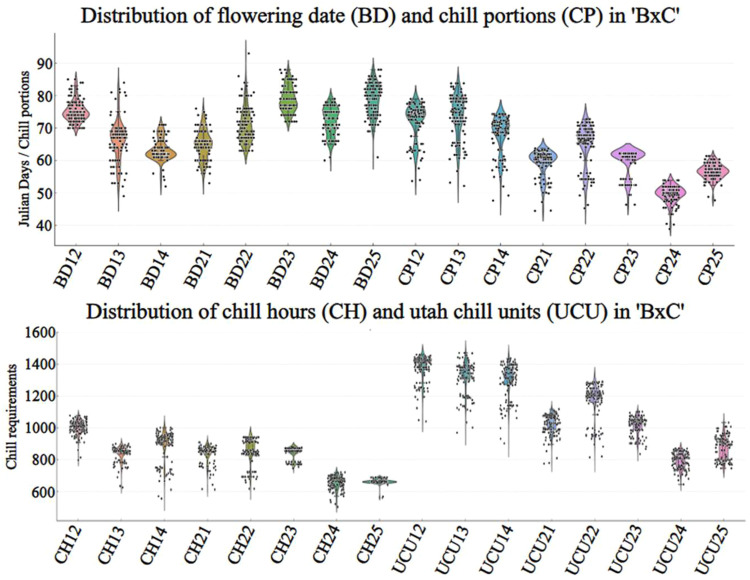
Distribution of flowering dates values and chilling requirement estimates (CH: Chill Hours, UCU: Utah Chill Units, and CP: Chill Portions) in the ‘BxC’ *P. armeniaca* population across multiple years. Each point represents an individual genotype, and the violin plots illustrate the density distribution for each season.

**Fig. 5:**
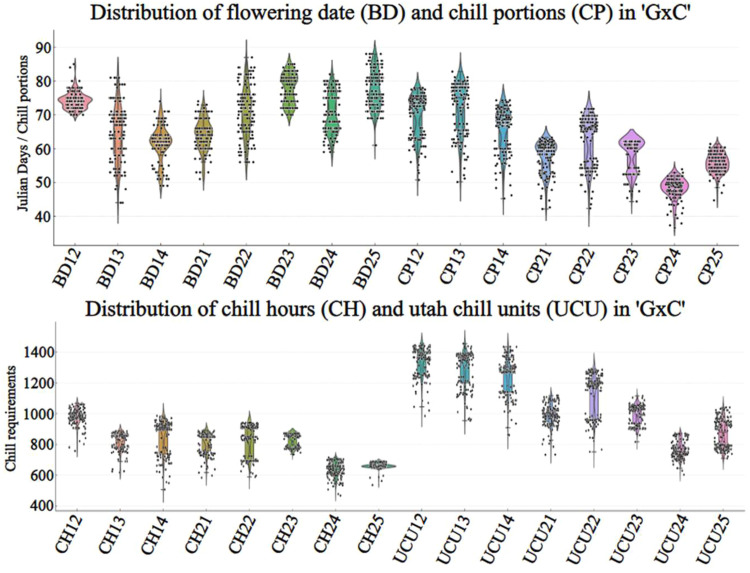
Distribution of flowering dates values and chilling requirement estimates (CH: Chill Hours, UCU: Utah Chill Units, and CP: Chill Portions) in the ‘GxC’ *P. armeniaca* population across multiple years. Each point represents an individual genotype, and the violin plots illustrate the density distribution for each season.

The outputs generated in this study have several potential applications, including QTL mapping, which relies on precise phenotypic data across segregating populations such as those evaluated in this study (BxC’ and ‘GxC’). Additionally, it facilitates the development of cultivar portfolios to guide growers in selecting varieties best suited to specific growing regions. As part of the validation, we present scatter plots showing the distribution of CR across numerous genotypes from both populations (Fig. 4, Fig. 5). These visualizations provide a valuable reference for future studies investigating the genetic basis of CR through integrated phenotypic and genotypic analyses, ultimately facilitating marker-trait association studies aimed at developing climate-resilient cultivars.

### Limitations

The main contribution of this study is the development of an R-based workflow for estimating CR across a large number of temperate fruit tree genotypes using PLS regression. Reliable CR estimates are a valuable resource for agronomists, breeders, and researchers working on dormancy-related topics. The applicability of the code was demonstrated with a case study on apricot trees, and the CR results are well aligned with our current understanding of CR variation in a segregating population derived from a low- and high-chill parental crosses. Notably, the CR estimate for one of the parents in the ‘GxC’ progeny (‘Currot’) was consistent with previous values obtained using the forcing method at the same experimental site [6]. However, the relatively limited number of flowering seasons in our dataset may constrain the robustness of the results. Expanding the dataset with additional phenological records could improve accuracy. Additionally, this workflow improves the comparability of CR studies by establishing a standardized criterion for defining the chill accumulation period. While PLS regression has proven effective in estimating CR when experimental data are not available, some inconsistencies have been reported when comparing previous studies [23,24]. In this sense, future research should focus on refining methods to accurately determine the end of dormancy, as well as improving chill models that better represent how different temperature regimes influence phenology.

## Ethics statements

Not applicable.

## CRediT author statement

**Ana M. Muñoz-Morales**: Conceptualization, Methodology, Software. **Germán Ortuño-Hernández**: Validity tests, Data curation. **Juan Alfonso Salazar**: Visualization, Investigation, Data curation. **Pedro Martínez-Gómez**: Supervision. **Jose A. Egea**: Software, Validation. **David Ruiz**: Supervision, Data curation. **Álvaro Delgado**: Conceptualization, Methodology, Writing- Reviewing and Editing.

## Acknowledgments

The authors acknowledge the support of the Spanish Ministry of Science and Innovation through the project "Apricot Breeding (APRIBRED)" (PID2022–137392OB-100). This research was also conducted within the framework of the AGROALNEXT programme, supported by the Ministry of Science and Innovation (MCIN), the European Union (NextGenerationEU, PRTR-C17.I1), and Fundación Séneca, funded by the Comunidad Autónoma de la Región de Murcia (CARM). Álvaro Delgado acknowledges support from the Spanish Ministry of Science and Innovation through a postdoctoral research grant (JDC2022–050,286-I), Juan Alfonso Salazar is supported by a ‘Ramón y Cajal’ postdoctoral contract (RYC2022–038,101-I), and Germán Ortuño-Hernández by a predoctoral fellowship (FPU21/03,563).

## Declaration of interests

The authors declare that they have no known competing financial interests or personal relationships that could have appeared to influence the work reported in this paper.

**Supplementary material *and/or* additional information [OPTIONAL]**

*If you do submit supplementary files, you are welcome to provide supporting details in this OPTIONAL section.*

## Data Availability

The complete code and associated datasets are freely available in a public repository (https://github.com/CEBASFruitBreed/R-workflow-ChillPLS)
